# Molecular basis of pathogenesis of postharvest pathogenic Fungi and control strategy in fruits: progress and prospect

**DOI:** 10.1186/s43897-021-00004-x

**Published:** 2021-06-16

**Authors:** Zhan-Quan Zhang, Tong Chen, Bo-Qiang Li, Guo-Zheng Qin, Shi-Ping Tian

**Affiliations:** 1 Key Laboratory of Plant Resources, Institute of Botany, The Innovative Academy of Seed Design, Chinese Academy of Sciences, Beijing, 100093 China; 2 University of Chinese Academy of Sciences, Beijing, 100049 China

**Keywords:** Pathogenic genes, Regulation mechanism, Postharvest disease, Control strategy, Fruit

## Abstract

The disease caused by pathogenic fungi is the main cause of postharvest loss of fresh fruits. The formulation of disease control strategies greatly depends on the understanding of pathogenic mechanism of fungal pathogens and control strategy. In recent years, based on the application of various combinatorial research methods, some pathogenic genes of important postharvest fungal pathogens in fruit have been revealed, and their functions and molecular regulatory networks of virulence have been explored. These progresses not only provide a new perspective for understanding the molecular basis and regulation mechanism of pathogenicity of postharvest pathogenic fungi, but also are beneficial to giving theoretical guidance for the creation of new technologies of postharvest disease control. Here, we synthesized these recent advances and illustrated conceptual frameworks, and identified several issues on the focus of future studies.

## Introduction

Fruits constitute an indispensable part of people’s dietary structure and are closely related to human health because of the rich nutrition, such as vitamins, minerals and antioxidants, etc. However, recent data show that about 30% of the production of fruits loses during the postharvest handing, distribution and storage stage annually worldwide (OECD, 2014). Although there are many factors leading to postharvest loss of fruit, decay caused by pathogenic fungi is the major cause. The main postharvest pathogenic fungi include *Botrytis cinerea*, *Penicillium* spp., *Monilinia* spp., *Alternaria alternata*, *Rhizopus stolonifer*, *Trichothecium roseum*, *Fusarium* spp*.*, *Colletotrichum* spp*.*, and so on. Among them, *B. cinerea* has been considered as the second most important plant pathogenic fungus (just follows *Magnaporthe oryzae*) because it is able to cause gray mold disease in various horticultural crops, resulting in over a billion dollars of losses every year in the world, and also serves as a model system to reveal molecular mechanism of pathogenicity of postharvest pathogens (Dean et al., 2012). In addition to causing quality deterioration and economic losses, some postharvest fungi also pose threat to human health, since some fungal genera, such as *Penicillium*, *Fusarium* and *Alternaria*, can produce mycotoxins, which are toxic to humans (Li et al., 2015; Sanzani et al., 2016).

According to the traditional view, most postharvest pathogenic fungi are typical necrotrophic pathogens, which kill the host cells by the secreted cell wall degrading enzymes or toxins directly, and then absorb nutrients from the dead cells (Tian et al., 2016). Interestingly, many postharvest pathogens, such as *A. alternata*, and *Colletorichum geoeosporioides* etc. can infect fruit at preharvest stage and remain quiescent for a long time in the process of growth and development, but initiate necrotrophic life style in ripening or senescing fruit (Prusky et al., 2013). Recent studies on the pathogenesis of postharvest pathogens demonstrate that these pathogenic fungi have more sophisticated interaction process with host than previously estimated. For example, *B. cinerea* can induce complex programmed cell death of host cells to facilitate its infection (Shlezinger et al., 2011). Meanwhile, the small RNA produced by *B. cinerea* can be secreted into host cells and inhibit the immune response of plants by using the local AGO proteins to promote the infection process (Weiberg et al. 2013).

In this review, we will briefly summarize the research progress on the pathogenic genes and regulation mechanism of pathogenicity of postharvest pathogenic fungi, and the control strategy of postharvest diseases in fruits.

## Molecular mechanism of pathogenicity

**Fig. 1:**
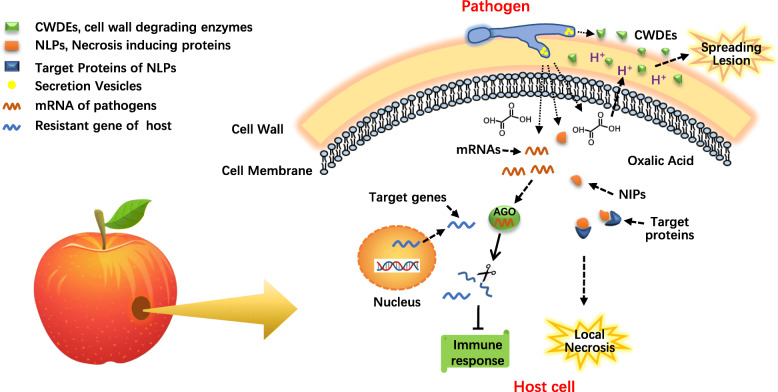
Pathogenic mechanism of postharvest pathogenic fungi

The occurrence of diseases is the process of the interaction between pathogenic fungi and fruit hosts. The process is more complex than previously estimated and includes some pathogenic genes and different mechanisms. Understanding the molecular basis of pathogenicity will shed light on the complicated mechanisms of the pathogenesis of postharvest pathogenic fungi (Fig. 1).

Various molecules, including secreted proteins, phytotoxic metabolites and small RNA, contribute to the infection process. At the early stage of infection, postharvest pathogenic fungi secrete necrosis-inducing proteins to induce the local necrosis of host cells for successful colonization. Subsequently, they secrete a large amount of cell wall degrading enzymes and secondary metabolites to promote spread of cell death. Small RNAs derived from pathogenic fungi can inhibit immune responses of host by hijacking the plant RNA interference system.

### Pathogenic genes of postharvest pathogens

#### Secreted proteins

During the infection progress, postharvest pathogenic fungi secrete a lot of proteins and metabolites to facilitate their colonization in the host. For example, in banana pathogen *F. proliferatum*, a total of 105 extracellular proteins could be induced by banana peel, and 40 of them were exclusively secreted in response to banana peel (Li et al., 2019b). The secreted hydrolytic enzymes can help pathogens invade host tissues by breaking down the physical barrier of plant, and further decompose plant tissues to provide necessary nutrients for the growth of pathogens. Cutinases, that decompose the peripheral physical barrier cuticle of host plant, have been considered to be an important virulence factor in some postharvest pathogenic fungi, such as *C. geoeosporioides* (Dickman and Patil 1986) and *Monilinia fructicola* (Lee et al., 2010). Plant cell wall, mainly composed of cellulose, hemicelluloses and pectin, is the important defensive barrier that the invasive pathogens encounter. A series of cell wall degrading enzymes (CWDEs) of the postharvest pathogenic fungi are involved in the degradation of host cell wall (Tian et al., 2016). The CWDE endopolygalacturonases (PGs) are critical virulence factors for the postharvest pathogenic fungi. There are six PGs in *B. cinerea*, and two of them (BcPG1 and BcPG2) are responsible for the full virulence (ten Have et al. 1998; Kars et al. 2005). Interestingly, among the six PGs, only BcPG1 and BcPG2 were the PGs identified in the early secretome, and they were ranked among the ten most abundant secreted proteins (Espino et al., 2010). Similarly, there are also two PGs (Pdpg1 and Pdpg2) involved in the pathogenesis of *Penicillium digitatum* (Vilanova et al., 2018). Pectin methylesterases (PMEs) can demethylate pectin and make it more prone to degradation by PGs. In *B. cinerea*, the deletion of PME gene *Bcpme1* led to significant decrease of the virulence (Valette-Collet et al. 2003). In addition, xylanase and cellobiohydrolase were also found to have significant influence on the pathogenicity of *B. cinerea* (Brito et al., 2006; Li et al., 2020). In postharvest pathogenic fungi, although more than 1000 proteins were predicted to enter the secretion pathway, the number of pathogenic factors identified in the extracellular proteins by genetic methods is limited (González et al., 2012). This may be attributed to the functional redundancy of these proteins with similar function. For example, there are 14 extracellular enzymes potentially involved in the degradation of cellulose, and 28 enzymes potentially involved in the degradation of pectin (González et al., 2016).

Considering that conidia are the main source of the infection of most postharvest pathogens, the massive secretion of CWDEs at the early stage of infection is unlikely. Necrosis-inducing proteins (NLPs) play an important role for the successful establishment of necrotrophic fungi at the initial phase of infection. In *B. cinerea*, NEP proteins (BcNEP1 and BcNEP2), ceratoplatanin protein (BcSpl1), xyloglucanase (BcXYG1) and BcIEB can produce local necrosis when applied to plants in an isolated form (Schouten et al., 2010; Frías et al., 2011; González et al., 2017; Zhu et al., 2017). Only knockout of BsSpl1 led to the significant decrease of virulence in *B. cinerea,* but the deletion of other NLPs had no effect on its virulence (Frías et al., 2011), indicating there are functional redundancy for necrosis-inducing proteins. Recently, two NEP proteins (Penlp1 and Penlp2) in *P. expansum* were also identified by Levin et al. (2019). Both of them showed necrosis-inducing activity, and deletion of *Penlp1*, but not *Penlp2*, resulted in reduced virulence of *P. expansum* on apple fruits (Levin et al., 2019). Some of the secreted virulence proteins can target the pathogenesis-related (PR) proteins of plant hosts and manipulate their resistance response. For instance, BcIEB1 can interact with the PR protein osmotin in plant and protects *B. cinerea* against their antifungal activity (González et al., 2017). These results indicate that secretory proteins play an important role in the pathogenicity of postharvest pathogenic fungi.

#### Phytotoxic metabolites

Pathogenic fungi can produce various phytotoxic metabolites with low molecular weight. The best studied phytotoxic metabolite in postharvest pathogens is botrydial in *B. cinerea*. Botrydial is produced in infected plant tissues, and can cause the withering of plant (Colmenares et al., 2002). A gene cluster has been identified to be responsible for the biosynthesis of botrydial, including at least two cytochrome P450 monooxygenase genes. Deletion of one of the cytochrome P450, *Bcbot1*, resulted in significant reduction of virulence (Siewers et al., 2005).

Other important phytotoxic metabolite in necrotrophic pathogens is organic acids. To some extent, organic acids are cofactor in infectious process, rather than a primary phytotoxic agent. Some postharvest pathogens, such as *B. cinerea*, *Sclerotinia sclerotiorum*, *P. expansum*, *P. digitatum* and *P. italicum*, assist the infection by acidifying the host tissue (Manteau et al., 2003; Prusky and Yakoby, 2003). Organic acids can enhance the activity of CWDEs by lowering the environmental pH value and induce the programmed cell death (Prusky and Lichter, 2008). *B. cinerea* and *S. sclerotiorum* decrease the host pH value by secreting a large amount of oxalic acid (Rollins and Dickman 2001; Manteau et al. 2003), whereas *Penicillium* spp. mainly secretes gluconic and citric acids to host cells (Prusky et al. 2004). In contrast, some other postharvest pathogens, like *C. gloeosporioides* and *A. alternata*, alkalize the host by secreting ammonia to promote the infection (Eshel et al., 2002), suggesting that the pH value of pulp tissue is related to virulence of postharvest pathogenic fungi.

#### Reactive oxygen species

Reactive oxygen species (ROS), including singlet oxygen (^1^O^2−^) superoxide anion (•O^2−^), hydroxyl radical (•OH) and hydrogen (H_2_O_2_), are small molecules with high oxidative activity, and usually produced as byproducts of metabolic processes in organisms (Heller and Tudzynski, 2011). A large amount of studies have indicated that ROS play an important role in the fruit-microbe interactions (Qin et al., 2007; Qin et al., 2011; Li et al., 2019a, b; Wang et al., 2019). On one hand, when fruits are attacked by pathogens, fruit cells can rapidly accumulate a large amount of ROS around the infectious site (Tian et al., 2013); On the other hand, ROS derived from pathogenic fungi also play a critical role during the interaction process (Tian et al., 2016). In fungi, NADPH oxidase complex (Nox) is the most important enzyme for ROS production. Nox is localized to plasma membrane or endoplasmic reticulum membrane and transports electrons through membranes to reduce oxygen molecule to •O^2−^ using NADPH as electron donor (Bedard et al., 2007). In *B. cinerea*, the two catalytic subunits, NoxA and NoxB, are involved in the different stages of infection, and the common regulatory subunit, NoxR, possesses the additive functions for NoxA and NoxB (Segmüller et al., 2008). Deletion of NoxR in *B. cinerea* led to reduced vegetative growth, conidiation, and impaired virulence on various hosts (Li et al., 2016). Furthermore, NoxR can regulate the protein abundant of 6-phosphogluconate dehydrogenase (6-PGD) and actin, which both have been proved to affect the development and pathogenicity of *B. cinerea* (Li et al., 2020). Some other subunits or inferred subunits of NOX, such as Bem1, Cdc24 and Rho, were also closely related to the pathogenicity of postharvest pathogenic fungi (Giesbert et al., 2014; An et al., 2015). Among them, Rho3 can also regulate the polar distribution of ROS in the hyphae, which is critical for the development and pathogenicity (An et al., 2015). In addition, we proved that one aquaporin protein, AQP8, could regulate the formation of infectious structure and virulence of *B. cinerea* via mediating the transmembrane transport of ROS (An et al., 2016).

#### Small RNAs

Small RNAs (sRNAs) are short regulatory nonconding RNAs that silence target genes with fully or partially complementary sequences (Baulcombe, 2004). They are produced by Dicer or Dicer-like endoribonucleases from double-stranded RNAs or single-stranded RNAs (Ghildiyal and Zamore, 2009). Mature sRNAs are first loaded into Argonaute (AGO) proteins, direct RNA-induced silencing complex (RISC) to target genes, and then induce gene silencing by guiding mRNA cleavage, translation inhibition or epigenetic modification (Baulcombe, 2004; Castel and Martienssen, 2013), which is called RNA interference (RNAi). RNAi plays an important role in regulating plant immunity against various pathogens (Seo et al., 2013), and pathogen-derived sRNAs also contribute to virulence of pathogens (Schmidtke et al., 2013). Recent studies indicated that *B. cinerea* can secrete small RNAs to the host cell to selectively silence plant genes involved in defense responses, by hijacking the plant RNA interference system. Weiberg et al. (2013) identified 73 sRNAs with the potential to silence immune genes of plant hosts in *B. cinerea* by deep sequencing. They found that these sRNAs could be pre-processed by the Dicer enzymes of *B. cinerea*, and once they were secreted to the host cells, they bind to plant argonaute protein to silence the specific target genes of host. Then, Cai et al. reported that plants send small RNAs in extracellular vesicles to fungal pathogen to silence virulence genes (Cai et al., 2018), suggesting the importance of small RNAs in the interactions between plant host and fungal pathogens.

### Regulatory pathways of pathogenic genes

#### Signal transduction

The interactions between pathogen and host are precisely regulated by signal transduction. Signaling cascades transmit signals across membranes to cytosol and nucleus, then the cellular response is arranged by the signals. Through genetic screening, many components of signal cascades have been proved to have important regulatory effects on the pathogenicity of postharvest pathogenic fungi (Schumacher, 2016). The cell surface receptor G-protein-coupled receptors (GPCRs) perceive environmental signals and relay them to intracellular signaling pathways. The mutation of a GPCR gene of *B. cinerea* led to slightly reduction in virulence (Schulze et al., 2004). BOS1, a histidine kinase receptor in cell membrane, is involved in osmoregulation, resistance to dicarboximide phenylpyrrole fungicides and virulence of *B. cinerea* (Viaud et al., 2006). The histidine kinase CpHK2 in *Claviceps purpurea* also regulates the spore germination, oxidative stress, and virulence (Nathues et al., 2007). Heterotrimeric G protein is an upstream component of signal cascades which can be directly regulated by GPCR. In *B. cinerea*, two α subunit genes of heterotrimeric G protein, *bcg1* and *bcg2*, were identified, and the deletion of these two genes led to a significant decline of virulence (Schulze et al., 2001; Döhlemann et al., 2006). The second messenger cAMP is involved in multiple processes in plant pathogenic fungi, including vegetative growth, conidiation, nutrient sensing and virulence (Kronstad, 1997). The adenylate cyclase regulates the intracellular cAMP levels and is responsible for the development and full virulence of *B. cinerea* (Klimpel et al., 2002). MAP kinase-controlled signaling pathway is highly conserved in eukaryotes. In several plant pathogens, MAP kinases are essential for the early phase of infection, specifically the penetration of plant surfaces (Solomon et al., 2005). Three MAP kinase genes, *Pdos2, PdSlt2* and *PdMpkB*, have been proved to regulate the pathogenicity of *P. digitatum* (Ma et al., 2016). The knockout mutant *△Pdos2* and *△PdSlt2* showed reduced virulence on citrus fruit, and *△PdMpkB* lost pathogenicity completely (De Ramón-Carbonell and Sánchez-Torres, 2017). The knockout of MAP kinase gene *bmp1* in *B. cinerea* also resulted in total nonpathogenicity (Zheng et al., 2000). Another MAP kinase gene *BcSAK1*, the homolog of the *Saccharomyces cerevisiae* HOG1, has been proved to play an important role in vegetative and pathogenic development of *B. cinerea*, because the *Δbcsak1* mutants failed to produce conidia and was unable to penetrate unwounded plant tissues (Segmüller et al., 2007). MAP kinase gene *BcMkk1* in *B. cinerea* can negatively regulate the biosynthesis of virulence factor oxalic acid through inhibiting phosphorylation of Per-Arnt-Sim (PAS) kinase BcRim15 mediated by kinase BcSch9 (Yin et al., 2018). By contrast, the hog1-like genes in other pathogenic fungi, such as *C. lagenarium* (Kojima et al., 2004) and *M. grisea* (Dixon et al., 1999), did not or slightly affect pathogenicity. Small G proteins (monomeric GTPases) function as molecular switches in the signal cascades and regulate a variety of biochemical reactions. Ras family GTPases Bcras1/2 and Rho family GTPases Bcrac/Bccdc42 are involved in the regulation of differentiation and virulence of *B. cinerea* (Kokkelink et al., 2011; An et al., 2015). Bccdc42 and Rho3 regulates differentiation and virulence of *B. cinerea* by affecting nuclear division, reducing conidial germination and penetration property (Kokkelink et al., 2011), and decreasing ROS accumulation in the hyphae tips (An et al., 2015).

#### Transcriptional regulation

Transcriptional regulation is an important regulatory mechanism in various biological processes. Transcriptional factors (TFs) can interact specifically with cis-acting elements in gene promoter region, and regulate the spatio-temporal expression of target gene. Son et al. systematically analyzed the phenotypes of 657 TF mutants of *F. graminearum*, and found that TFs play crucial role in regulating the development, stress response, toxin synthesis and pathogenicity (Son et al., 2011). There are abundant of TF coding genes in the genome of postharvest pathogenic fungi, but only a small number of them have been functionally characterized to date. The STE family TF Ste12, a downstream component of MAPK signal cascade, can regulate the penetration process of *B. cinerea, P. digitatum* and *P. expansum* on tomato leaf, citrus fruit and apple fruit, respectively (Schamber et al., 2010; Vilanova et al., 2016; Sánchez-Torres et al., 2018). The calcineurin-responsive Crz1 and Reg1 have been proved to be involved in the development and pathogenesis of *B. cinerea* and *P. digitatum* (Schumacher et al., 2008; Michielse et al., 2011). Moreover, we proved that the MADS-box family TF Bcmads1 regulates the virulence of *B. cinerea* by affecting the protein secretion process and sclerotia formation via mediating the expression of light responsive genes (Zhang et al., 2016). These results demonstrate the important role of transcriptional regulation in the growth, development and virulence of postharvest pathogenic fungi.

#### Secretion regulation

Extracellular enzymes and metabolites are important “weapons” for postharvest pathogenic fungi to attack fruit hosts. The secretion process is precisely regulated. The Rab family small GTPase involved in the vesicle docking and fusion, plays a central role in the secretory pathway (Novick and Zerial 1997). The knockout of SEC4-like Rab/GTPase gene (*CLPT1)* in *C. lindemuthianum* led to a lethal phenotype (Dumas et al., 2001), suggesting that CLPT1 is necessary for the delivery of proteins to extracellular environment and critical for the differentiation of infectious structures. Our previous studies showed that Rab8-like protein Bcsas1 in *B. cinerea* regulated the polar transport of secretion vesicles. Deletion of *Bcsas1* inhibited the secretion of some critical virulence factor, such as polygalacturonase and xylanase, eventually leading to the decrease of pathogenicity of *B. cinerea* (Zhang et al., 2014). In addition, the Rab family GTPase Bcsec14 and Bcsec31 regulated by the TF Bcmads1 are also related to the secretion of extracellular protein and are required for pathogenesis of *B. cinerea* (Zhang et al., 2016). A protein Blistering1 containing DnaJ domain has recently been shown to modulate the virulence of *P. expansum* via affecting vesicle-mediated protein secretion, and the insertion mutant of *Blistering1* failed to secrete various CWDEs and had significantly reduced capacity to degrade apple tissue (Jurick et al., 2020).

#### Environmental regulation

The virulence of pathogenic fungi is regulated not only by intercellular factors, but also by various environmental factors, particularly by ambient pH value, which has significant effects on the development and pathogenicity of pathogenic fungi (Manteau et al., 2003). Usually, different organs of plant have different pH levels, for example, fruits show lower pH values (about 3.3–4.5), while leaves, stems and roots exhibit higher pH value (about 5.8–6.5). With the senesce of fruit, the pH value gradually increases because respiration firstly consumes organic acids, which have a significant impact on the virulence of postharvest pathogenic fungi. Our previous study demonstrated that the environmental pH value in vitro impacted the growth and development of pathogenic fungi via affecting the pH value in the cell of fungal pathogen, and the conidial germinability of *P. expansum* was significantly inhibited when pH value is 2 or 8, in which intercellular protein synthesis and folding were impaired (Li et al., 2010). Additionally, some postharvest pathogenic fungi can infect fruit at preharvest stage and remain quiescent for a long time in developing fruit, but show symptom in ripening or senescing fruit, indicating that these pathogens can adapt to a wide range of pH values. Based on the study of the effect of different ambient pH levels on the secretome component of *B. cinerea*, we found that lower pH level (pH 4, represents the pH value of fruit) induced the secretion of protein related to proteolysis, and higher pH level (pH 6, represents the pH value of leaves) induced more cell wall degrading enzymes (Li et al., 2012), implying that *B. cinerea* has the ability to adjust protein profile of secretome to respond to different ambient pH value of fruit host.

During the interaction process, the pathogenic fungi can also positively adjust the ambient pH level to establish the optimal infection conditions by secreting acid or alkaline substances, which were considered as phytotoxic metabolites (Prusky and Lichter, 2008). Fungi have evolved a sophisticated system to respond to the ambient pH. The pH response system is regulated by Pal signaling pathway, which was well characterized in *Aspergillus nidulans* (Peñalva et al., 2008). Seven genes have been identified in Pal pathway, including *pacC*, *palA*, *palB*, *palC*, *palF*, *palH* and *palI* (Peñalva et al., 2008). PacC is a pH-dependent global transcription factor and has been widely characterized in postharvest pathogenic fungi. Our results indicated that *BcPacC* in *B. cinerea* could be significantly induced by high pH level, suggesting that it is involved in the response to ambient pH (Li et al., 2012). *PePacC* affected the virulence of *P. expansum* by regulating the expression of pathogenic factors PeCRT (calreticulin) and PeSAT (sulfate adenylyltransferase) (Chen et al., 2018). Meanwhile, *pacC* has also been found to be responsible for the full virulence in other postharvest pathogens, such as *C. gloeosporioides* (Alkan et al., 2013), *A. alternata* (Eshel et al., 2002), and *F. oxysporum* (Caracuel et al., 2003). These results indicate the influence of pH value on the virulence of postharvest pathogenic fungi.

## Postharvest disease control strategy

**Fig. 2:**
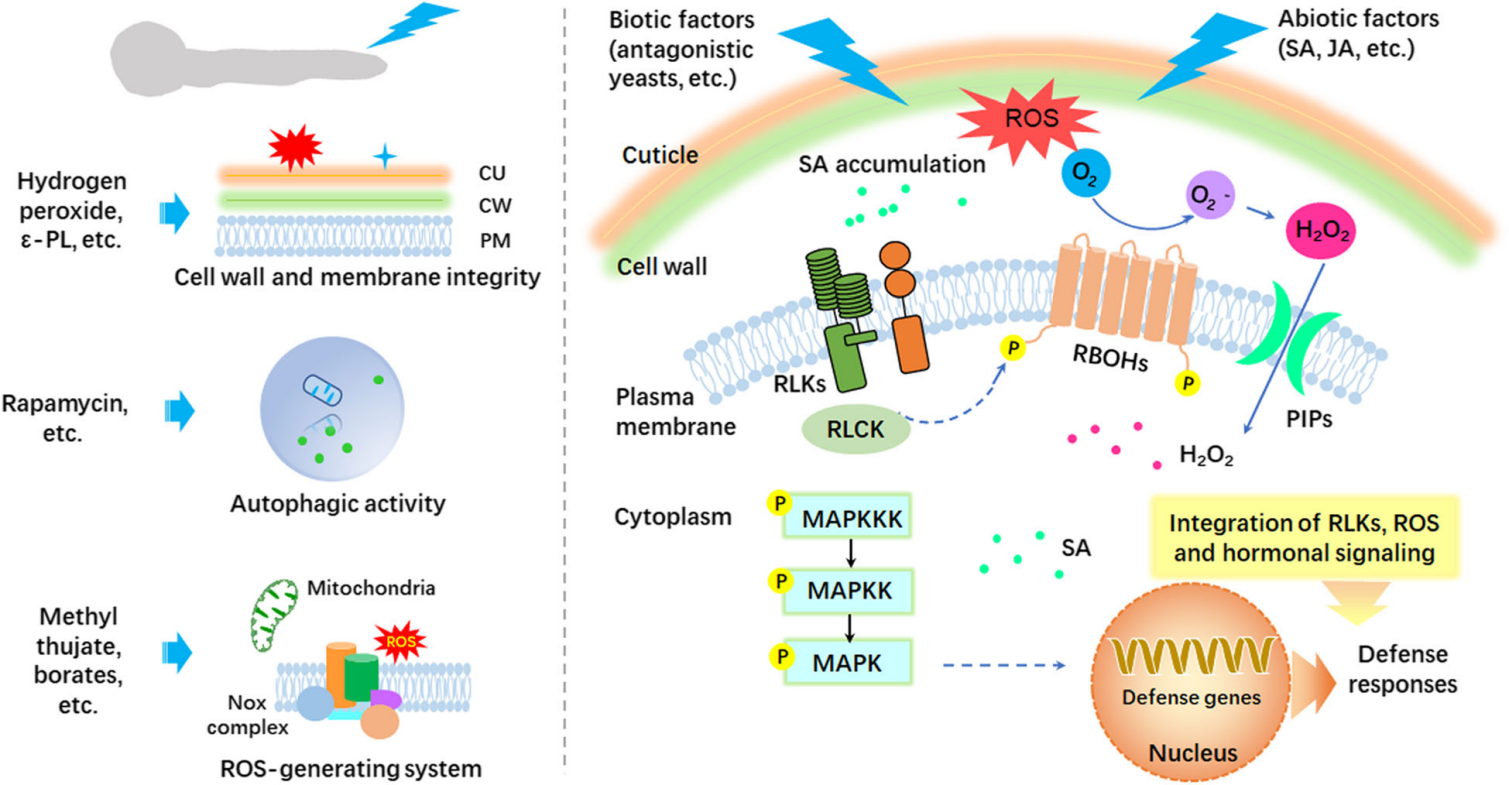
Strategies for controlling postharvest pathogenic fungi

Several strategies have been employed to control postharvest diseases of fruits and vegetables, such as low temperature storage, controlled atmosphere storage, treatments with chemicals, heat treatment and biological control. Due to the requirements for specific instruments and certain limitations for some methods, treatment with synthetic chemicals and low temperature storage are currently widely applied under practical conditions (Tian et al., 2016). The fruit-pathogen interactions are closely related to developmental stages of fruit and diversified environmental conditions (Tian et al., 2016). Control strategies of postharvest disease include two parts. One is the direct action on pathogenic fungi via impacting the pathogenic genes; the other is the induction of fruit resistance to resist the invasion of pathogenic fungi using biotic and abiotic factors (Fig. 2).

Control over postharvest diseases may be realized by directly affecting the genes related to pathogenesis or inducing fruit resistance to resist the invasion of pathogenic fungi. These inhibitory actions may target cell wall and membrane integrity, autophagic activity, ROS-generating system, and other targets in fungal cells. Biotic and abiotic factors may integrate RLKs, ROS and hormonal signaling to induce defense responses. Alternatively, they may function as PAMPs to induce pattern-trigged immunity by RLKs, RLCK and MAPK cascade, thus activating the expression of defense genes.

CU: cuticle; CW: cell wall; PM: plasma membrane; PAMP: pathogen-triggered molecular pattern; RLK: receptor-like kinase; RLCK: receptor-like cytoplasmic kinase; MAPK: mitogen-activated protein kinase; SA: salicylic acid; JA: jasmonic acid; PIP: plasma membrane intrinsic proteins.

### Targeted regulation of postharvest pathogens

Due to the importance of ROS equilibrium systems in fungi, they have become one of the first-choice targets for many exogenous antifungal substances. For example, borates have been proved to be effective in controlling many postharvest pathogens, such as *P. expansum* and *C. gloeosporioides*, by inhibiting antioxidant catalase and glutathione S-transferase (Qin et al., 2010; Shi et al., 2011). Hydrogen peroxide can induce ROS generation in mitochondria to cause oxidative damage of mitochondrial proteins and led to the collapse of mitochondrial membrane potential and cell death of *P. expansum* (Qin et al., 2011). Methyl thujate, a terpene substance from conifer species, has inducing effect on the expression of *Nox* genes in pathogenic fungi and lead to the excessive accumulation of ROS in fungal cells, thus inhibiting the pathogenicity (Ji et al., 2018; Ma et al., 2020). Alternatively, autophagic activity is also a newly reported target. Rapamycin substantially inhibited mycelial growth of *B. cinerea*, which was attributed to the modulation in autophagic activity and the down-regulation in the expression of key genes (*bctor*, *bcatg1*, *bcatg8* and *bcatg14*) involved in autophagy (Ma et al., 2019). These results were further confirmed by monodansylcadaverine (MDC) staining and transmission electron microscopy. Aside from the functions for activating antioxidative capacity of host cells, luteolin, a flavonoid substance, is efficient for suppressing mycelial growth of *B. cinerea* and *P. expansum* (Liu et al., 2020). Coincidently, several key genes (*pepatE*, *pepatK*, *pevelB*, *pelaeA*, *pepatL* and *peveA*) responsible for patulin biosynthesis in *P. expansum* were down-regulated upon the exogenous application with luteolin, further indicating that luteolin is promising to be developed as an alternative agent for controlling fungal pathogen and mycotoxin production.

### Induction of resistance in fruit hosts

Plants usually develop resistance to resist the infection when they are attacked by pathogenic fungi. These resistant responses mainly involve hypersensitive responses, production of phytoalexins, papillae formation and toxin degradation (Zhou and Zeng, 2018). Phenylpropanoid metabolic pathway contributes to nearly all aspects of plant responses towards biotic and abiotic stimuli by producing a wide range of phenylpropanoid compounds (Vogt, 2010). In general, there are two resistant response pathways in plant, one is the systemic acquired resistance (SAR) induced by abiotic factor, the other is the induced systemic resistance (ISR) stimulated by biotic factor. Salicylic acid (SA) has been well recognized as a crucial signaling molecule involved in the activation of plant defense responses. Exogenous application of SA can induce resistance of sweet cherry fruit against infection by *P. expansum* (Chan et al., 2008). In addition, *Pichia membranefaciens,* an antagonistic yeast, can effectively control postharvest disease caused by *P. expansum* in peach fruit via upregulating antioxidant enzymes and pathogenesis-related (PR)-proteins (Chan et al., 2007). Jasmonic acid (JA), another signaling molecule, has crucial roles in inducing resistance against pathogenic fungi. Accumulating studies have shown that MeJA induces disease resistance in tomato and loquat fruit against *B. cinerea* and *C. acutatum* via affecting pathogenesis-related genes and defense-related enzymes as well as the production of specific secondary metabolites (Cao et al., 2008; Yu et al., 2009; Zhu and Tian, 2012). Notably, transcriptomic analyses have proven that the genes involved in ethylene signaling, jasmonate signaling and MYB–domain transcription factor family are over–represented in the resistant *Malus sieversii* genotype (Ballester et al., 2017).

## Prospect

Growing evidences have implied that the interactions between postharvest pathogenic fungi and fruit hosts are much more sophisticated. It is generally considered that pathogenic fungi may have a brief biotrophic phase prior to the onset of necrotrophic cycle. During this brief process, there must be a complex “dialog” between the pathogenic fungi and fruit hosts. The extracellular proteins (or metabolites) are the most important weapon employed by the pathogenic fungi against the immune response of fruit hosts. Therefore, the profile of extracellular proteins is a good candidate for unraveling the unknown interaction mechanisms between postharvest pathogenic fungi and fruit hosts in the future investigation. The identification of more and more pathogenic genes will provide theoretical basis for formulating targeted control strategies for postharvest diseases of fruits. It will be an important research direction in the future to achieve effective control of postharvest diseases through targeted regulation of key pathogenic genes.
